# Artificial Intelligence as a Tool in the Diagnosis of Bladder Cancer: A Narrative Review

**DOI:** 10.7759/cureus.96958

**Published:** 2025-11-16

**Authors:** Sundas Ishtiaq, Khalid Farouk

**Affiliations:** 1 Department of Urology, Fauji Foundation Hospital and Foundation University Medical College, Islamabad, PAK

**Keywords:** artificial intelligence, cystoscopy, histopathology, machine learning, urinary bladder neoplasms

## Abstract

Artificial intelligence (AI) is emerging as a transformative tool in the diagnosis of bladder cancer, offering the potential to enhance accuracy, consistency, and early detection. This narrative review aimed to summarize and critically appraise recent developments in AI applications across diagnostic modalities, based on studies identified through PubMed, Scopus, and Google Scholar up to April 2025. Evidence shows that deep learning algorithms applied to cystoscopy improve lesion detection, including subtle flat and early-stage tumors that may be overlooked by conventional assessment. In histopathology and cytology, AI systems contribute to grading, classification, and identification of malignant features with accuracy comparable to expert pathologists. Integration of AI with urinary biomarkers and genomic data further supports personalized risk prediction and molecular characterization. Additionally, AI-driven clinical decision support tools assist in prognosis, treatment planning, and post-therapy surveillance. While current findings underscore AI’s promising role in improving diagnostic precision and workflow efficiency, its clinical adoption requires addressing issues of data quality, algorithm transparency, and ethical governance. Future research should focus on developing explainable and validated models through multicenter collaborations between clinicians and data scientists to facilitate safe and reliable integration of AI into routine bladder cancer diagnosis.

## Introduction and background

Bladder cancer (BC) remains one of the most common malignancies worldwide, ranking as the 10th most prevalent cancer globally [1]. According to GLOBOCAN 2018, approximately 500,000 new cases of BC are diagnosed annually, with a significant proportion of the burden occurring in men [1,2]. The male-to-female ratio for incidence is nearly 3:1, reflecting both biological differences and variations in exposure to established risk factors such as smoking and occupational carcinogens [2]. In addition to its incidence, BC is associated with one of the highest recurrence rates among solid tumors, with up to 70% of non-muscle-invasive cases relapsing after initial treatment due to the field-change effect, persistence of residual tumor cells, and incomplete resection during initial therapy [1,2]. This frequent recurrence necessitates lifelong surveillance through repeated cystoscopies, urine cytology, and imaging, thereby creating a substantial diagnostic and economic burden. The need for continuous monitoring and accurate differentiation between recurrence, progression, and benign lesions increases diagnostic complexity, underscoring the demand for more sensitive and objective tools.

Despite advances in cancer detection, the current diagnostic gold standards, cystoscopy, urine cytology, and histopathological examination, continue to present major limitations. Cystoscopy, while allowing direct visualization of the bladder mucosa, is invasive and costly, and its diagnostic performance depends heavily on operator expertise [3]. Urine cytology, though highly specific for high-grade disease, suffers from low sensitivity, particularly for detecting low-grade tumors [3,4]. Histopathological assessment provides critical information on tumor grade and stage, but is constrained by inter-observer variability, subjectivity, and time consumption [4]. Together, these limitations hinder early and accurate detection, which is crucial since BC often presents as a non-muscle-invasive disease but carries a risk of progression to invasive or metastatic stages that are more difficult and costly to treat [3,4]. Hence, the combined challenge of high recurrence and diagnostic limitations highlights an urgent need for innovative solutions capable of improving diagnostic precision and efficiency.

In recent years, artificial intelligence (AI) has emerged as a promising adjunctive tool in healthcare, with the potential to overcome several of these diagnostic challenges [5]. AI encompasses a range of computational methods capable of learning from large datasets, recognizing complex patterns, and generating predictive insights [5,6]. Within AI, machine learning (ML) algorithms and deep learning (DL) architectures, such as convolutional neural networks (CNNs), have demonstrated remarkable success in tasks like medical image analysis, cytology interpretation, and genomic classification [6-8]. In urologic oncology specifically, AI has shown promise in enhancing cystoscopic lesion detection [9-13], improving the accuracy and reproducibility of histopathological and cytological evaluation [14-17], and integrating biomarker and genomic data for individualized diagnostic profiles [18,19].

While many of AI’s advantages remain theoretical, such as potential cost reduction, workflow optimization, and improved accessibility, several demonstrated benefits have been reported in early studies, including enhanced lesion detection, diagnostic consistency, and reduced observer variability [10,11]. Unlike human interpretation, which may vary across observers and institutions, AI models offer standardized, reproducible assessments that can potentially reduce diagnostic errors [7,8]. Moreover, AI has the capacity to handle multimodal datasets, incorporating imaging, pathology, and molecular data simultaneously, thereby advancing precision medicine [20,21]. In addition, explainable AI (XAI) models are beginning to address concerns about transparency, clinical applicability, and ethical implementation [22-24].

XAI refers to methods that make model decisions interpretable to clinicians by highlighting which features or image regions contribute most to a prediction. For instance, in cystoscopic image analysis, heatmaps generated by AI can visually mark suspicious areas that influenced the algorithm’s decision, allowing urologists to verify and trust the model’s reasoning. Such transparency enhances clinician confidence and supports integration of AI as a decision-support tool rather than a black-box system [22,23]. Nevertheless, challenges remain regarding the integration of AI into routine clinical practice. These include data quality and heterogeneity, the need for large-scale multicenter validation, regulatory and ethical issues surrounding algorithm transparency, and the potential unintended consequences of over-reliance on automated systems [25-27]. Furthermore, despite an increasing number of FDA-cleared AI-based medical devices [28], few have yet been widely implemented in urologic oncology, highlighting the gap between technological development and real-world application.

Given the rapid progress of AI technologies and their expanding role in cancer diagnostics, it is essential to review their current applications and evaluate both strengths and limitations in the context of BC. The present narrative review synthesizes available evidence on the diagnostic applications of AI in BC, focusing on four domains: (i) cystoscopy and imaging, (ii) histopathology and cytology, (iii) urinary biomarkers and genomics, and (iv) clinical decision support (CDS). These domains were selected because they encompass the major diagnostic pillars of BC care, ranging from initial lesion detection and tissue evaluation to molecular characterization and integrated clinical decision-making.

By examining the current state of the field and addressing challenges to implementation, this review aims to provide insights into how AI may contribute to earlier detection, improved diagnostic accuracy, and better patient outcomes in BC [7,24,29]. Despite growing research interest, evidence on AI in BC diagnosis remains fragmented, with studies differing in methodology, scope, and clinical applicability. Most data originate from high-income countries, while research from South Asia, including Pakistan, is scarce despite the significant disease burden and resource constraints. A comprehensive synthesis is therefore needed to consolidate existing knowledge, identify gaps, and provide clinicians and researchers with an updated perspective. This review not only summarizes global advances but also emphasizes their potential relevance and adaptability in resource-limited healthcare systems. It aims to guide future research directions, foster multicenter collaboration, and support the integration of AI into BC diagnostics, where early detection and cost-effective solutions are most urgently required. Specifically, it addresses the question: How has AI been applied across different diagnostic modalities in BC, and what evidence supports its clinical validity, reliability, and potential for routine integration?

## Review

Methodology

This narrative review was conducted to evaluate the current role of AI in the diagnosis of BC. A comprehensive literature search was performed using four major electronic databases, PubMed, Scopus, Google Scholar, and Embase, from inception to April 2025. The search strategy combined Medical Subject Headings (MeSH) and free-text terms to maximize sensitivity. The main keywords included “artificial intelligence”, “machine learning”, “urinary bladder neoplasms”, “cystoscopy”, and “histopathology”. Boolean operators (AND/OR) were applied in various combinations. Filters were used to restrict results to human subject studies published in the English language, directly relevant to diagnostic applications. References of retrieved articles were also screened to identify additional eligible studies.

Inclusion criteria were studies evaluating the use of AI, ML, or DL in any diagnostic aspect of BC; involving human subjects or human-derived data (imaging, histopathology, cytology, or biomarkers); and published in peer-reviewed English-language journals between January 2015 and April 2025. Exclusion criteria included studies limited to treatment response, prognosis, or non-urological cancers, as well as grey literature, preprints, conference abstracts, and animal studies.

The initial search retrieved 312 articles. After removing 74 duplicates, 238 articles underwent title and abstract screening, of which 168 were excluded for focusing on therapeutic or prognostic applications rather than diagnosis. The full texts of the remaining 70 studies were reviewed, and 42 met the inclusion criteria for final analysis. These comprised original research articles, systematic reviews, and narrative reviews providing an overview of current evidence.

Screening and data extraction were conducted independently by two reviewers, with discrepancies resolved by discussion. Each included study was appraised for methodological quality, sample size, data source reliability, validation strategy, and reporting of key performance metrics such as accuracy, sensitivity, specificity, and area under the curve (AUC).

For synthesis, studies were thematically categorized into four domains of AI application: (i) cystoscopy and imaging algorithms for lesion detection and characterization, (ii) histopathology and cytology-computer-assisted image analysis for grading and staging, (iii) urinary biomarkers and genomics-predictive models integrating molecular and genomic data, and (iv) CDS, AI-based diagnostic aid, and risk stratification tools. This structured approach enabled systematic appraisal of the evidence, highlighting strengths, limitations, and future research directions.

**Figure 1:**
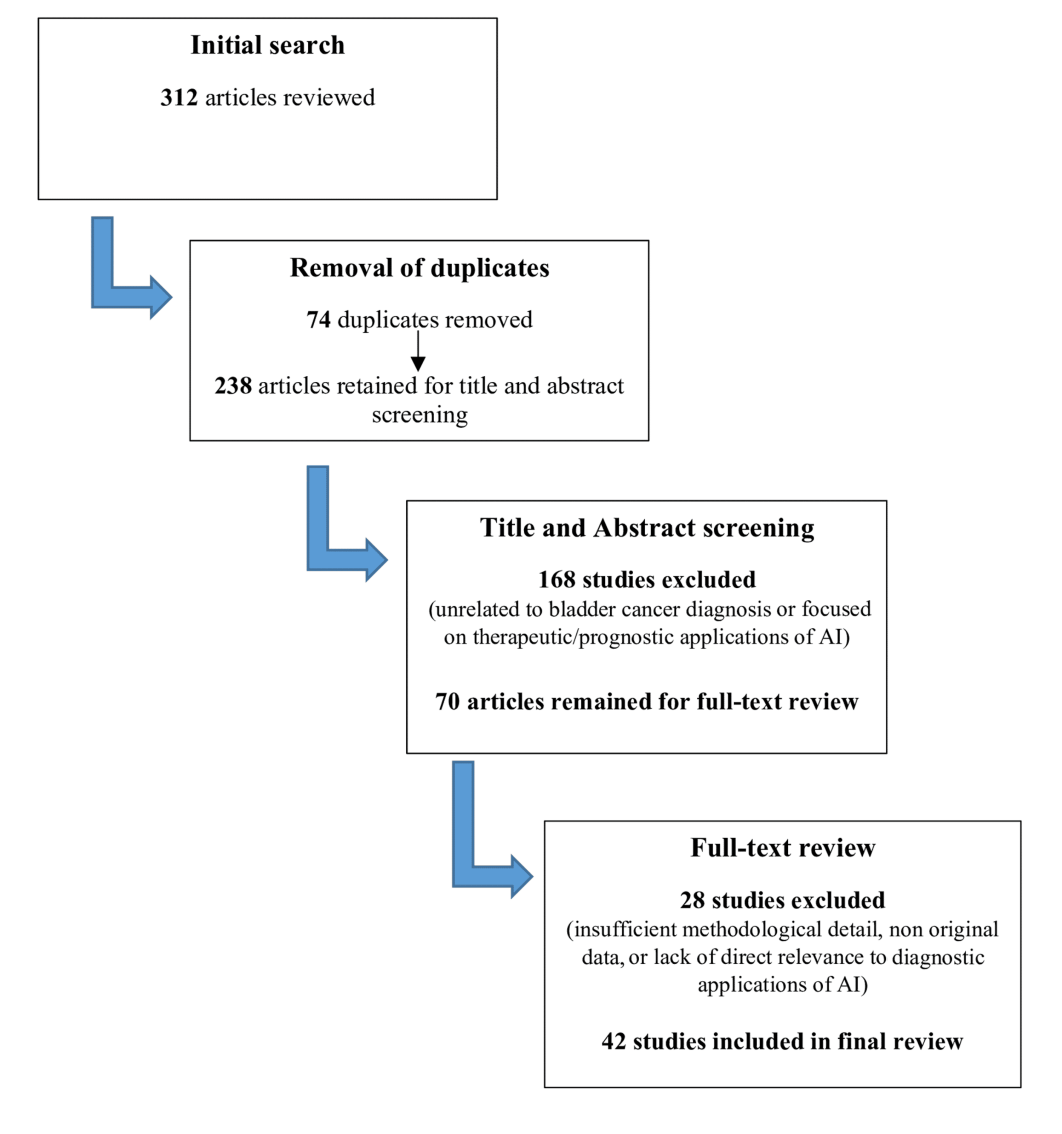
Study selection process AI: artificial intelligence

Given the heterogeneity in study designs, data types, and outcome measures, a narrative synthesis was chosen over meta-analysis to allow integrative discussion of methodological trends, diagnostic relevance, and evidence gaps across these domains. The study selection process is shown in Figure 1.

AI in cystoscopy and imaging

Cystoscopy remains the cornerstone of BC diagnosis and surveillance, recommended as the gold standard by the European Association of Urology (EAU) guidelines [3]. However, conventional white-light cystoscopy (WLC) has well-documented limitations, including difficulty in detecting flat lesions such as carcinoma in situ (CIS), variable sensitivity for small papillary tumors, and dependence on operator expertise [3,10]. Although adjunctive techniques such as narrow-band imaging (NBI) have improved visualization, they still suffer from substantial inter-observer variability and false negatives [10,29]. AI, particularly DL approaches using CNNs, has shown significant promise in addressing these shortcomings.

By training on large, annotated cystoscopic image datasets, AI systems are capable of distinguishing benign from malignant lesions with high accuracy and identifying subtle mucosal abnormalities that may be overlooked by human observers [29,30]. For instance, Shkolyar et al. demonstrated that a CNN-based algorithm enhanced the detection of bladder tumors during WLC, particularly flat and small lesions that are frequently missed in standard practice [10]. Similarly, Wu et al., in a recent systematic review and meta-analysis, reported that AI-assisted cystoscopy significantly improved diagnostic sensitivity without compromising specificity, highlighting its value as an adjunct to conventional endoscopy [9]. Emerging prospective validation studies strengthen the clinical utility of these systems. Iwaki et al. conducted a multicenter validation trial and found that DL models achieved consistent performance across different institutions, supporting their generalizability in real-world settings [11]. More recently, Chang et al. demonstrated in a prospective clinical trial that real-time AI-assisted cystoscopy reduced the rate of missed diagnoses and improved the selection of biopsy sites, thereby enhancing diagnostic yield and optimizing patient management [13]. In parallel, Hu et al. explored AI-based tumor mapping, showing that automated delineation of tumor margins can assist in surgical planning and improve staging accuracy [12].

From a technical perspective, AI-assisted cystoscopy offers several advantages: real-time feedback, standardization of lesion recognition, and the potential to reduce inter-observer variability [29,30]. Importantly, by supporting tumor localization and mapping, AI systems may facilitate more precise resections and follow-up strategies, contributing to personalized management pathways [31]. AI integration into cystoscopy represents a paradigm shift in BC diagnostics. While still requiring large-scale validation and regulatory approval, current evidence strongly suggests that AI-assisted cystoscopy enhances lesion detection, supports accurate staging, and ultimately has the potential to improve patient outcomes. Table 1 gives a summary of AI applications in cystoscopy and imaging for BC.

**Table 1:** Summary of AI applications in cystoscopy and imaging for bladder cancer diagnosis WLC: white-light cystoscopy; AUC: area under the curve; AI: artificial intelligence

Study (Authors, year)	AI Method	Dataset/Inputs	Outcome Measures	Key Findings
Shkolyar et al., 2019 [10]	Convolutional Neural Network (CNN)	White-light and narrow-band cystoscopy images	Sensitivity, specificity, AUC	CNN improved bladder tumor detection with an AUC of 0.98, outperforming standard cystoscopy, especially for flat and small lesions.
Wu et al., 2019 [9]	Deep Learning Model	Multicenter cystoscopic imaging dataset (WLC)	Sensitivity, specificity, diagnostic accuracy	AI-assisted cystoscopy significantly increased diagnostic sensitivity without loss of specificity across diverse centers, and enhanced the detection of subtle lesions.
Iwaki et al., 2023 [11]	Deep Neural Network	Multicenter cystoscopic images (Hunner lesion recognition)	Accuracy, sensitivity, specificity	Demonstrated generalizable performance across institutions, supporting reliable real-world application of AI in cystoscopy.
Chang et al., 2023 [13]	Real-time AI-assisted system (Deep Learning)	Prospective cystoscopic video	Missed lesion rate, detection accuracy, biopsy guidance	Real-time AI system reduced missed diagnoses and improved selection of biopsy sites, enhancing diagnostic yield.
Hu et al., 2019 [12]	Deep Learning for image reconstruction	1,620 prospective cystoscopy images	Reconstruction quality, segmentation accuracy	Enabled automated bladder lesion mapping/reconstruction, assisting visualization and potentially improving surgical planning.

AI in histopathology and cytology

Histopathology is central to the grading and staging of BC, guiding both prognosis and treatment strategies [4]. The advent of digital pathology has enabled the conversion of glass slides into high-resolution whole-slide images (WSIs), which can be analyzed using ML and DL algorithms [14]. These approaches facilitate automated tumor grading, highlight high-risk histological features, and improve diagnostic reproducibility, thereby reducing inter-observer variability that has long challenged traditional pathology [14,15]. Several studies have demonstrated that AI can enhance the diagnostic accuracy of histopathological assessment in urothelial carcinoma. For example, Komura and Ishikawa outlined how ML techniques are being applied to tissue image analysis, with CNN-based models excelling in feature extraction and tumor classification [14]. Beyond BC, Bulten et al. showed that AI assistance significantly improved the Gleason grading of prostate biopsies, underscoring the cross-specialty potential of these tools to standardize pathological interpretation [15]. More recently, Campanella et al. developed a clinical-grade DL framework that successfully analyzed WSIs across multiple cancer types, paving the way for scalable AI applications in pathology [32]. Notably, Chen et al. [33] and Coudray et al. [34] reported that DL algorithms could not only classify cancer subtypes with high accuracy but also predict underlying mutational patterns directly from histology, an innovation with major implications for precision oncology. Furthermore, Steiner et al. demonstrated that combined AI-pathologist assessment improved diagnostic performance over either modality alone, highlighting the role of AI as a supportive rather than replacement tool [35].

Cytology, particularly urine cytology, remains an important adjunct in BC diagnosis and surveillance. However, its diagnostic accuracy is limited by low sensitivity for low-grade tumors, despite high specificity for high-grade disease [16,17]. Recent advances in DL have substantially improved performance in this domain. Li et al., in a study, concluded that AI-driven cytology systems enhance malignant cell detection and consistently outperform conventional manual methods [16]. Similarly, Liu et al. found that DL models improved the sensitivity of urinary cytology without compromising specificity, particularly for high-grade urothelial carcinoma [17]. The incorporation of AI into urine cytology workflows thus offers the potential to bridge diagnostic gaps, providing earlier detection and more reliable monitoring. Importantly, studies such as those by Campanella et al. [32] and Coudray et al. [34] reinforce that AI in cytology and pathology is not only accurate but also scalable to real-world clinical environments. AI has demonstrated substantial promise in both histopathology and cytology of BC by enhancing accuracy, reducing variability, and enabling novel predictive insights. With further validation and integration into routine workflows, AI is poised to become an indispensable adjunct to human expertise in uro-oncologic diagnostics. Tables 2, 3 give the summary of AI applications in histology and cytology for BC diagnosis, respectively.

**Table 2:** Summary of AI applications in histopathology for bladder cancer diagnosis CNN: convolutional neural network; WSI: whole-slide image; TCGA: The Cancer Genome Atlas; AUC: area under the curve; ML: machine learning; AI: artificial intelligence

Study (Authors, year)	AI Method	Dataset/Inputs	Outcome Measures	Key Findings
Komura & Ishikawa, 2018 [14]	Machine Learning & CNN-based image analysis	Digital WSIs from various tumor histologies	Feature extraction, tumor classification	Demonstrated how ML and CNNs can analyze WSIs for automated tumor grading, improved feature extraction, and reduced inter-observer variability.
Bulten et al., 2021 [15]	Deep Learning for biopsy grading	WSIs of prostate biopsies	Accuracy, inter-observer agreement	Showed that AI-assisted grading significantly improves consistency and accuracy—supporting cross-specialty generalizability to bladder pathology.
Campanella et al., 2019 [32]	Weakly-supervised Deep Learning	44,732 WSIs across multiple cancer types	Slide-level classification accuracy	Developed a clinical-grade DL system capable of analyzing WSIs at scale with high diagnostic accuracy, supporting real-world deployment in digital pathology.
Chen et al., 2021 [33]	Deep Learning for WSI analysis	Histology slides for cancer subtype classification	Classification accuracy, mutation prediction	Demonstrated that Deep Learning can classify cancer subtypes and predict underlying mutational signatures directly from pathology slides—advancing precision oncology.
Coudray et al., 2018 [34]	CNN for subtype and mutation prediction	TCGA lung cancer WSIs	Sensitivity/specificity, mutation prediction AUC	Showed that CNNs can detect actionable mutations and classify histological subtypes from morphology alone, supporting the concept of mutation prediction from tissue in bladder cancer contexts.
Steiner et al., 2020 [35]	Hybrid AI–pathologist workflow	WSIs from multiple cancer types	Accuracy, error reduction	Demonstrated that AI + pathologist collaboration improves diagnostic performance beyond either alone—supporting AI as an adjunct, not a replacement.

**Table 3:** Applications in cytology for bladder cancer diagnosis WSI: whole-slide image; CNN: convolutional neural network; AI: artificial intelligence

Study (Authors, year)	AI Method	Dataset/Inputs	Outcome Measures	Key Findings
Li et al., 2025 [16]	AI-driven cytology system	Digital urine cytology slides	Sensitivity, specificity, malignant cell detection	Showed that AI-based urinary cytology significantly improves malignant cell detection and consistently outperforms conventional manual evaluation.
Liu et al., 2022 [17]	Deep Learning cytopathology model	Urinary cytology images	Sensitivity, specificity	Demonstrated improved sensitivity—especially for high-grade urothelial carcinoma—without compromising specificity.
Campanella et al., 2019 [32]	Deep Learning for whole-slide classification	Large-scale WSI datasets	Diagnostic accuracy	Supports scalability of AI, applicable to cytology workflows in large-volume pathology labs.
Coudray et al., 2018 [34]	CNN for morphologic + genomic prediction	WSIs	Classification accuracy, mutation prediction	Reinforces the potential for AI to augment cytology by linking morphology to molecular signatures.

AI in urinary biomarkers and genomics

Molecular and urinary biomarkers are increasingly recognized as valuable tools in the diagnosis and prognostication of BC. Established assays such as FGFR3 and TERT promoter mutations, and urinary protein-based markers like NMP22, have demonstrated utility; however, their clinical adoption is limited by variability and reproducibility issues [18]. With the rapid expansion of genomic, transcriptomic, proteomic, and metabolomic datasets, interpreting this complexity requires computational methods capable of identifying clinically meaningful patterns [19-21]. AI offers a powerful solution by integrating heterogeneous biomarker data and constructing predictive models that reflect tumor heterogeneity. For instance, Robertson et al. [19] and Tan et al. [21] showed that AI-driven molecular classification can stratify urothelial carcinoma into distinct subtypes with prognostic and therapeutic implications. Bellmunt et al. similarly demonstrated the prognostic value of computational genomic predictors [20]. AI has also advanced biomarker discovery, uncovering molecular signatures that are not easily detectable with conventional analysis. Liao et al. [36] identified mutational signatures associated with prognosis, while Kamoun et al. [37] used integrative computational approaches to establish a consensus molecular classification of muscle-invasive BC. Complementary studies by Eriksson et al. [38] and Hurst et al. [39] revealed gene regulatory networks and metabolic pathways underlying disease subtypes, whereas Warrick et al. [40] emphasized the role of AI in capturing intratumoral heterogeneity to refine risk stratification. By linking urinary biomarkers with genomic data, AI enables not only improved diagnostic accuracy but also individualized prediction of recurrence, treatment response, and long-term survival [18,36]. Its capacity to uncover novel molecular signatures highlights its potential to drive precision medicine in BC, ultimately guiding risk-adapted surveillance and treatment strategies. Table 4 summarizes AI applications in urinary biomarkers and genomics.

**Table 4:** AI applications in urinary biomarkers and genomics BC: bladder cancer; AI: artificial intelligence; TCGA: The Cancer Genome Atlas; ML: machine learning;

Biomarker/Genomic Feature	AI/Computational Method	Key Findings	Clinical Relevance	Reference
FGFR3, TERT promoter mutations, NMP22	AI-enhanced multimodal integration of urinary and genomic biomarkers	Improved diagnostic accuracy when combined with AI analysis	Potential for non-invasive diagnosis and recurrence monitoring	[18]
Multi-omic data (DNA, RNA, protein)	Computational integration of TCGA datasets	Identified molecular subtypes of muscle-invasive BC	Stratifies patients for targeted therapies	[19]
Genomic predictors of outcome	Systematic review + ML-based predictors	Genomic signatures associated with prognosis and treatment response	Guides personalized therapy	[20]
Urothelial carcinoma subtypes (n=2411 tumors)	Meta-cohort analysis using AI clustering	Four molecular subtypes with distinct clinical outcomes	Risk stratification and therapy selection	[21]
Mutational signatures	AI-driven genomic characterization	Novel mutational profiles linked to outcomes	Prognostic markers for patient management	[36]
Muscle-invasive BC classification	Consensus computational classification	Six consensus subtypes validated across cohorts	Standardized classification for clinical trials	[37]
Gene regulatory networks	Network analysis with AI	Defined subtype-specific regulatory patterns	Insights into tumor biology and therapeutic targets	[38]
Metabolic profiles in non-invasive BC	AI-based genomic clustering	Identified subtypes with distinct metabolic signatures	Supports subtype-specific prognostication	[39]
Intratumoral heterogeneity	AI-driven molecular subtype analysis	Identified coexistence of multiple subtypes within tumors	Explains treatment resistance and recurrence	[40]

AI in clinical decision support

AI-driven CDS systems synthesize diverse clinical inputs-cystoscopy findings, histopathology, biomarkers, imaging, and molecular profiles-into actionable recommendations for clinicians [22,23]. Unlike traditional risk calculators, these models leverage ML and DL algorithms trained on large datasets that incorporate tumor stage, grade, genetic alterations, and patient history, thereby enabling individualized care pathways [22]. Obermeyer and Emanuel [22], for example, highlighted the role of predictive analytics in anticipating disease progression, while Senders et al. [23] emphasized AI’s capacity to assist physicians by integrating multimodal data for complex decision-making. Reddy et al. further demonstrated how AI-enabled healthcare delivery can support treatment stratification, optimize therapy selection, and improve patient outcomes by reducing delays and minimizing human error [24].

Moreover, integration with electronic health records (EHRs) allows AI systems to generate real-time alerts, such as reminders for surveillance cystoscopies, risk stratification for recurrence or progression, and recommendations for adjuvant therapy [41-44]. Miotto et al. reported that DL-based models outperform conventional statistical approaches in predicting clinical trajectories, especially when trained on longitudinal patient records [43]. Similarly, Rajkomar et al. showed that AI can achieve expert-level prediction of hospital outcomes across diverse clinical settings [42]. In BC, AI-based CDS tools have the potential to guide oncologists on whether patients with high-grade non-muscle invasive disease may benefit from early radical cystectomy, or whether intravesical therapy would suffice, by analyzing genomic signatures and prior treatment response [18,21,39]. Additionally, molecular classification studies [37-40] can be harnessed by CDS systems to stratify patients into molecular subtypes with distinct therapeutic and prognostic implications.

Despite these advances, challenges remain in the translation of AI models into routine urological practice. Concerns include the explainability of predictions [45,46], unintended consequences such as overreliance on algorithms [47], and issues of generalizability due to training datasets not reflecting diverse populations [25,41,48,49]. Regulatory frameworks, such as the Consolidated Standards of Reporting Trials-Artificial Intelligence extension (CONSORT-AI) guidelines [50], aim to improve the reporting, validation, and clinical integration of AI-enabled CDS tools. AI-driven CDS systems represent a transformative tool in BC management, offering personalized, data-driven recommendations that can enhance clinical accuracy, streamline workflows, and improve patient outcomes. While their promise is significant, widespread adoption requires overcoming challenges of transparency, validation, and integration into clinical practice.

Limitations and ethical considerations

Despite substantial progress in AI for BC diagnosis, multiple limitations and ethical challenges hinder its clinical translation.

The performance of AI models in BC diagnosis depends heavily on the quality, size, and diversity of training datasets. Many systems are developed using single-institution or homogeneous cohorts, which limits their external validity and applicability across diverse populations. This is particularly concerning in BC, where variations in prevalence, tumor morphology, and imaging protocols may influence diagnostic accuracy. Kelly et al. emphasized that bias in training data can perpetuate or even exacerbate healthcare disparities, highlighting the need for multicenter and demographically representative datasets [25].

DL algorithms often function as “black boxes,” generating outputs without transparent reasoning. Ribeiro et al. demonstrated that explainability is crucial for users to understand and trust AI predictions, especially in sensitive domains like oncology [26]. Similarly, Amann et al. [46] and Kelly and Young [48] stressed that models must provide interpretable outputs aligned with clinical reasoning to ensure accountability and clinician acceptance. Without transparency, clinicians may remain reluctant to adopt AI in high-stakes contexts such as cancer diagnostics.

AI systems must also address concerns related to patient privacy, informed consent, and fairness across demographic subgroups. Char et al. underscored that equitable AI requires robust performance across age, gender, and ethnic categories to avoid reinforcing structural inequities [27]. Compliance with international data protection laws, including General Data Protection Regulation (GDPR) and Health Insurance Portability and Accountability Act (HIPAA), remains essential to safeguard confidentiality [27,28]. Benjamens et al. reported that although the FDA has cleared over 60 AI-based devices, many approvals lack long-term validation, raising concerns about premature clinical deployment [28].

The absence of standardized frameworks for validation and reporting remains a critical barrier to clinical adoption. Kelly et al. highlighted that most AI studies are retrospective, with limited large-scale or prospective validation, thereby reducing confidence in their clinical utility [25]. To address this gap, Liu et al. introduced the CONSORT-AI extension, aimed at improving transparency in AI clinical trial reporting, though widespread adoption is still at an early stage [50]. Regulatory uncertainty, therefore, continues to slow integration into practice despite rapid technological advances.

Beyond technical challenges, the unintended consequences of AI adoption must also be considered. Louis et al. cautioned that overreliance on automated systems could erode clinical judgment and critical decision-making skills [44]. Strickland further reported that IBM Watson for Oncology (IBM Corp., Armonk, New York, United States) failed to meet expectations due to inconsistent recommendations and insufficient real-world validation, serving as a reminder of the risks of overpromising AI capabilities without adequate evidence [49].

In summary, while AI holds transformative potential for BC diagnosis, challenges related to dataset bias [25], interpretability [26,46,48], ethical safeguards [27,28], and regulatory clarity [25,50] must be addressed before widespread implementation. Robust multicenter datasets, transparent and explainable models, and interdisciplinary collaboration will be essential to ensure safe, equitable, and effective integration into clinical practice.

Future directions and opportunities

AI in BC diagnosis is poised for significant advancement, with several promising opportunities on the horizon. Multi-modal integration, combining imaging, genomics, biomarkers, and EHR data, can enhance diagnostic accuracy and predictive performance [18,22,36]. Real-time assistance through AI-guided cystoscopy enables on-the-spot lesion detection, improving intraoperative decision-making and diagnostic precision [10,29,31]. Furthermore, the integration of genomic and molecular profiles with clinical data supports personalized medicine, allowing for tailored treatment strategies based on individual tumor characteristics [18,23,41]. Finally, expanding the deployment of AI diagnostic tools to underserved regions can improve global accessibility and help reduce disparities in BC care [7,8,27].

Recent advances in multi-modal data integration are particularly promising, as no single diagnostic modality captures the full biological complexity of BC. Robertson et al. [19] and Kamoun et al. [37] demonstrated that molecular subtyping of urothelial carcinoma can stratify patients into prognostic categories with distinct therapeutic responses. When combined with imaging and biomarker data, AI models can achieve more robust predictions of disease progression and recurrence [36,38,40]. This integrative approach may serve as the foundation for precision diagnostics, moving beyond morphology-based diagnosis toward comprehensive biological profiling [18,21].

Another emerging opportunity lies in real-time AI applications during cystoscopy. Shkolyar et al. [10] and Chang et al. [13] showed that DL algorithms can highlight suspicious lesions intraoperatively, enhancing urologists’ detection accuracy and reducing missed diagnoses. As Wu et al. noted in their study, AI-assisted cystoscopy significantly improves sensitivity without compromising specificity, suggesting its utility in routine clinical workflows [9]. Widespread adoption of such systems could shorten diagnostic timelines, decrease inter-observer variability, and provide immediate decision support at the point of care [29-31].

Finally, the ethical imperative of equitable deployment cannot be overlooked. Topol [7] and Char et al. [27] emphasized that AI has the potential to democratize access to high-quality diagnostics, especially in low-resource settings where expert pathologists or advanced cystoscopy tools are scarce. By leveraging cloud-based platforms and low-cost computational infrastructure, AI could extend advanced diagnostic capabilities globally, thereby narrowing disparities in BC care [7,8].

## Conclusions

This review highlights that AI is emerging as a valuable tool for improving BC diagnosis across cystoscopic imaging, histopathology, urinary biomarkers, and genomic profiling. Evidence from existing studies suggests that AI can enhance accuracy, reduce inter-observer variability, and support earlier detection when integrated into conventional diagnostic pathways. Importantly, in regions such as Pakistan, where diagnostic resources are limited and disease burden is high, AI-assisted tools could help bridge gaps in expertise and access, thereby improving patient care.

Nonetheless, most available studies are constrained by small datasets, a lack of external validation, and limited applicability across diverse populations. Future research should prioritize multicenter collaborations, standardized evaluation, and cost-effective solutions to enable broader clinical adoption. With these refinements, AI is positioned to complement existing diagnostic modalities and contribute significantly to better outcomes for patients with BC.
